# The Impact of Frequent Alarm Exposure on Intensive Care Nurses—A Mapping of the Current State of Knowledge

**DOI:** 10.1155/ccrp/3865004

**Published:** 2026-11-04

**Authors:** Frida Bråberg, Johan Vesterlund, Åsa Roxberg, Maria Emilsson

**Affiliations:** ^1^ Intensive Care Unit NU Hospital Group Region Västra Götaland Trollhättan SE-461 85 Sweden; ^2^ Department of Health Sciences University West Trollhättan SE-461 86 Sweden hv.se

**Keywords:** alarm fatigue, impact, intensive care, intensive care nurse, monitoring

## Abstract

**Background:**

The caring environment in an intensive care unit is more intense than anywhere else in the hospital. The responsibilities of the intensive care nurse include operating medical equipment and continuously monitoring and evaluating the patient’s health status. Great demands are placed on being able to prioritize and be vigilant as situations can quickly become critical. To facilitate these demands, each medical device has an alarm system that warns if parameters can be life‐threatening. Alarms have been shown to have an impact on the nurse, but a mapping of the knowledge area is needed to compile the multifactorial impact that alarms can have.

**Aim:**

The aim of this study was to map the current state of knowledge regarding how alarms in an intensive care context affect intensive care nurses.

**Method:**

A scoping review that contains articles using both quantitative and qualitative methods with the aim of mapping the current area of knowledge.

**Results:**

One category, Impact of Alarms Exposer on Nurses and Patient Care Delivery, and four subcategories emerged through the analysis: “Adverse physical effects,” “Psychological vulnerability,” “Negative effects on the work performance,” and “Patient safety at risk.”

**Conclusion:**

Today’s technological advances in medicine, and more specifically in patient monitoring, mean that we can monitor patients to a greater extent. This leads to an increased number of alarms and a work environment characterized by more alarms. The conclusion is therefore that alarms have a multifactorial impact on the physical and mental health and work performance of intensive care nurses.

## 1. Introduction

The condition of patients admitted to an intensive care unit (ICU) is characterized by dysfunction in one or more vital organ systems, resulting in a critical illness associated with a high risk of life‐threatening complications. Consequently, the care of critically ill patients requires continuous monitoring of vital parameters, including heart rhythm, blood pressure, body temperature, oxygen saturation, and respiratory rate.

To ensure accurate and safe monitoring, medical devices are preprogrammed with specific alarm thresholds designed to promptly alert healthcare professionals to deviations from normal physiological values. In the ICU setting, multiple alarms from various medical devices are frequently present, and intensive care nurses are often responsible for several patients simultaneously connected to such equipment. This may result in concurrent alarms from different patients, requiring nurses to prioritize between them. This situation has the potential to affect both patients and intensive care nurses. The extent to which intensive care nurses are affected by such alarm exposure is the focus of this study.

The Swedish Intensive Care Registry [1] illustrates the scope of the high‐technology environment in which patients’ health status is continuously monitored using medical equipment such as infusion pumps, ventilators, and monitoring systems. These devices generate both auditory and visual alarms, which may be burdensome [2].

The ICU environment is often described as acoustically disharmonious. Limited physical space in ICU rooms further exacerbates this issue, as the facilities are not designed to accommodate the increasing number of medical devices required in modern intensive care. Intensive care nurses report exposure to numerous disturbing and irritating sounds generated by medical equipment, which contribute to fatigue, a desire for silence after work, and avoidance of social activities during leisure time [3].

Continuous exposure to clinical alarms and monitoring alert systems can contribute to alarm fatigue among nurses [4]. Previous research has demonstrated the impact of alarms on nurses’ health. Cho et al. [5] reported that nurses experience moderate to high levels of alarm fatigue. Similarly, Jeong and Kim [6] found that nurses reported high levels of alarm fatigue over the preceding 6 months, which was strongly associated with perceived stress. Continuous exposure to frequent and prolonged alarms contributes to stress, anxiety, and increased alarm fatigue, posing a potential threat to patient safety [7]. Furthermore, repeated exposure to alarms lacking clinical significance may lead to desensitization, increasing the risk that nurses fail to respond appropriately to clinically relevant alarms [8].

The aim of this study was to map the current state of knowledge regarding how alarms in an intensive care context affect intensive care nurses.

## 2. Methods

### 2.1. Design

Given that the aim of this study was to map the current state of knowledge regarding the impact of alarms on intensive care nurses, a scoping review based on the framework proposed by Arksey and O’Malley [9] was selected. This approach is appropriate for providing an overview of existing evidence and identifying knowledge gaps related to alarm fatigue among intensive care nurses.

The scoping review methodology follows five stages: (1) identifying the research question, (2) identifying relevant studies, (3) selecting studies for inclusion, (4) charting the data, and (5) collating, summarizing, and reporting the results.

The first stage, identifying the research question, determines the search strategy appropriate for the area of interest. A broad conceptualization of “impact” was adopted to capture various aspects of intensive care nurses’ experiences of alarms. This approach is consistent with Arksey and O’Malley’s [9] recommendation to maintain a broad perspective in order to avoid excluding relevant information.

The second stage involved identifying relevant studies. The search was conducted using three approaches: database searches, reference list screening, and manual selection of studies deemed highly relevant to the study aim.

The third stage involved selecting studies that addressed the research aim. To enhance credibility and reliability, the screening process was conducted using the software Rayyan [10], which facilitates blinded and independent article selection. In this study, articles were selected independently by the authors to minimize the risk of selection bias.

The analysis was inspired by the manifest content analysis described by Graneheim and Lundman [11] (see the Analysis section for further details).

The script was translated from Swedish to English using ChatGPT as a translation tool. The translation was thoroughly reviewed by the research team and adapted as needed to ensure accuracy and natural language flow. The use of artificial intelligence (AI) was in accordance with the journal’s policy.

### 2.2. Sample

This study aimed to map the current state of knowledge regarding the impact of alarms on intensive care nurses. Therefore, the sample was only included to nurses working in ICUs.

Inclusion criteria were studies published in English, focusing on nurses’ experiences, addressing the study aim with alarms as the primary focus, and published between 2015 and 2025. In accordance with Arksey and O’Malley’s [9] methodology, studies were included regardless of methodological quality, provided they were deemed highly relevant to the research question.

Studies not conducted in intensive care settings or primarily focused on alarm management rather than impact were excluded. The databases used for the systematic search were CINAHL, PubMed, PsycINFO, and Cochrane, as recommended by Polit and Beck [12] for nursing research.

### 2.3. Data Collection

An initial exploratory search was conducted during the project planning phase to determine whether alarms had previously been studied. This approach helped refine the search strategy and broaden the understanding of the research problem, as suggested by Polit and Beck [12].

The search terms that yielded the most relevant results were alarm fatigue, ICU, ICU or critical care, and nurses’ experience, perspective, view, or attitude. These terms were combined using the Boolean operators “AND” and “OR,” a strategy recommended by Polit and Beck [12] to initially capture a broad range of studies and subsequently refine the search.

Inclusion criteria were peer‐reviewed articles written in English and available in full text. A supplementary search was conducted in PubMed, excluding the “Free Full Text” filter from the search strategy.

Studies not addressing the research aim, focusing on patients, or involving individuals under 18 years of age were excluded.

The exploratory search was repeated at the start of the study to identify newly published articles, resulting in an increased number of relevant studies. This led to adjustments in inclusion and exclusion criteria as well as the addition of new search terms. The final search terms used in each database are presented in the supporting information, Appendix 1.

Search terms were adapted for each database to ensure a comprehensive overview of the available evidence. Although the use of subject headings was intended, no relevant terms were identified that generated appropriate results. An additional search including the term impact resulted in one further article being included.

The final set of articles was imported into Rayyan [10]. Titles and abstracts were screened and categorized as “Include,” “Maybe,” or “Exclude.” Articles marked as “Maybe” or “Exclude” by one or both authors were resolved through discussed and reviewed in full text to determine inclusion or exclusion (see Figure 1).

**FIGURE 1:**
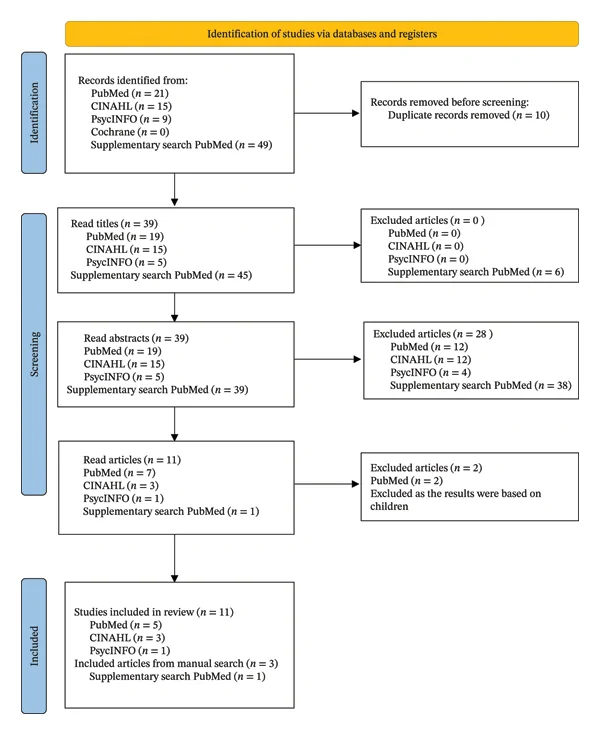
PRISMA flow diagram for study selection [13].

Manual searches were performed using the reference lists of included articles, as well as articles marked as “Mayby” in Rayyan, to find any relevant studies that had not been identified in the systematic search. This process resulted in the inclusion of an additional four articles.

### 2.4. Analysis

For this study, a manifest content analysis was employed, inspired by the approach described by Graneheim and Lundman [11]. Manifest content analysis focuses on describing what the text explicitly states, that is, the visible and obvious components of the content, in contrast to latent content analysis, which involves interpreting the underlying meaning of the text.

One of the most fundamental decisions in content analysis is the selection of the unit of analysis, which should reflect a broad variation of the phenomenon under investigation. Meaning units are defined as constellations of words, sentences, or paragraphs that are related through their content and context.

The texts, that is, the results sections of the included articles, were read repeatedly to ensure familiarity, after which meaning units were identified.

The identified meaning units were systematically extracted and condensed, a process that involved shortening the text while preserving its core meaning. The condensed meaning units were subsequently abstracted into codes. The coding process aimed to capture the essential content of each meaning unit and facilitated a more structured and analytical approach to the data.

Codes with similar content and meaning were continuously compared and grouped into subcategories. The subcategories were then further organized into broader category that described the manifest content of the material. The category thus represented recurring patterns and variations within the findings of the included studies. Throughout the analysis, meaning units, codes, subcategories, and categories were continuously compared to ensure internal homogeneity and external heterogeneity. The emerging category and subcategories were repeatedly reviewed against the original material to ensure that the synthesis remained grounded in the findings of the included studies and that no significant information was lost. Through this iterative process, the findings were synthesized into a coherent description of the phenomenon under investigation.

Disagreements between the reviewers regarding data extraction, coding, and categorization were resolved through discussion and repeated examination of the original study findings. Consensus was reached through an iterative review process, whereby interpretations were continuously compared with the source material to ensure a trustworthy and accurate representation of the data (see Table 1).

**TABLE 1:** Example of analysis process.

Meaning unit	Condensed meaning	Cod	Subcategory	Category
Most nurses revealed that they experienced headaches	Alarms cause headaches	Headache	Adverse physical effects	Impact of Alarms Exposure on Nurses and Patient Care Delivery

### 2.5. Preunderstanding

Authors Frida Bråberg and Johan Vesterlund have clinical experience in general ward‐based care as well as intensive care settings where alarms are highly prevalent. Author Åsa Roxberg has primarily worked in academia as a teacher and researcher. Author Maria Emilsson has several years of experience as a specialist nurse in intensive care and currently works as a senior university lecturer.

## 3. Results

The aim of this study was to map the current state of knowledge regarding how alarms in an intensive care context affect intensive care nurses.

The findings of this study are based on 13 articles (see supporting information for an overview of the study in Appendix 2), the majority of which employed quantitative methods. The analysis resulted in one main category and four subcategories (Table 2).

**TABLE 2:** The overview shows the analysis process and the four subcategories that created the category.

Subcategories	Category
Adverse physical effects	Impact of Alarms Exposure on Nurses and Patient Care Delivery
Psychological vulnerability	
Negative effects on work performance	
Patient safety at risk	

This category Impact of Alarms Exposer on Nurses and Patient Care Delivery describes the bodily responses that nurses reported as effects of alarms. The four subcategories’ physical and psychological impact influences on the work performance and influences on the patient safety of alarms on intensive care nurses.

### 3.1. Adverse Physical Effects

Excessive noise levels may negatively affect nurses’ physical health [14, 15]. The frequent and intense sound of alarms contributes to pronounced physical exhaustion among nurses. Alarms act as stressors that not only demand rapid physical responses but also lead to fatigue and physical tiredness [16, 17].

Furthermore, constant high‐frequency alarm sounds have been described as a source of physical discomfort, including headaches [17]. Karahan et al. [16] reported that 75% of participating nurses perceived that the frequency and intensity of alarms increased their physical exhaustion.

### 3.2. Psychological Vulnerability

Nurses report that a substantial proportion of noise in ICU settings originates from alarms. These alarms contribute not only to increased noise levels but also to sensory overload [14, 17, 18].

Alarms require continuous mental alertness. Persistent alarm sounds have been reported to cause distress, anxiety, and fatigue among nurses [16, 17]. Alarm fatigue has also been associated with burnout, secondary traumatic stress, and compassion fatigue among intensive care staff [19].

The intensity and frequency of alarms require nurses to remain constantly attentive, contributing to increased stress and psychological strain [14, 17, 18]. Nurses who are already physically or psychologically exhausted, or who experience burnout or compassion fatigue, appear more susceptible to alarm fatigue. These predisposing factors increase vulnerability to the negative effects of continuous alarm exposure.

Compassion fatigue may impair nurses’ ability to respond appropriately to alarms [20]. Similarly, Şen et al. [21] identified a correlation between compassion fatigue and alarm fatigue, suggesting a reciprocal relationship. Prolonged exposure to alarms has also been shown to reduce patience, further exacerbating stress‐related symptoms [22].

Frequent alarms may therefore result in desensitization, increasing the risk that critical alarms are missed or responded to late [14, 15, 18]. Carelli et al. [22] reported that the majority of nurses experienced a sense of indifference when alarms occurred repeatedly. Excessive noise levels may also negatively affect nurses’ psychological well‐being [14].

### 3.3. Negative Effects on Work Performance

Alarms also affect nurses’ by working overload and ability to perform their duties [14, 17, 18]. High workload may lead to alarms being ignored or reduced attentiveness [14, 15, 18, 23, 24]. False alarm noise has been likened to a “the wolf is coming” phenomenon, which may lead to indifference [15].

Continuous alarm disturbances create barriers to completing essential tasks, leading to frustration and a sense of inadequacy [18].

Advancements in modern technology have increased monitoring capabilities but also the frequency of false alarms, creating additional distractions. The increased number of monitored parameters contributes to a higher volume of alarms and makes it more difficult to identify clinically significant, life‐threatening alarms [15, 24]. A large majority of nurses (93%) reported that alarm fatigue led to silencing or ignoring alarms [24].

Additionally, 86.9% of nurses reported that alarms interfered with their work capacity, and decreased alertness was observed as alarm fatigue increased [16]. Attention to alarms was also reported to decrease during visiting hours [22].

Nurses’ productivity was moderately reduced due to the frequency and disruptive nature of alarms [17]. Time spent managing alarms—particularly false alarms—increases workload, reduces trust in equipment, decreases attentiveness, and disrupts patient care, often resulting in alarms being turned off [15, 16, 25].

Increased workload has been associated with delayed response times to alarms, thereby reducing work performance and trust in alarm systems [24]. High alarm noise levels may be intrusive and stress‐inducing, making it difficult for nurses to perform their tasks effectively [14].

Frequent false and disruptive alarms reduce response rates. Alarms may also be triggered in empty patient rooms or when patients are disconnected, which has been shown to reduce response rates by up to 50% [24].

On average, alarms occur between 150 and 400 times per day, the majority of which are false [24]. This significantly increases workload and directly affects nurses’ work performance. Karahan et al. [16] reported that 81.3% of nurses perceived alarms as contributing to increased workload.

### 3.4. Patient Safety at Risk

Alarm‐related fatigue contributes to risks in patient safety [4]. Several incidents have been reported where patient deaths were associated with alarm‐related issues [26]. In one case, alarms were turned off postoperatively and not reactivated, resulting in unrecognized respiratory distress and subsequent patient death. In another case, a patient died due to unrecognized hypoxia because the monitoring equipment was turned off [26].

Lewandowska et al. [24] reported cases where nurses failed to respond to alarms indicating low heart rate due to alarm fatigue, resulting in fatal outcomes. Excessive noise levels may negatively affect patient safety, as important alarms may be overlooked [14, 16].

Bani Hani and Abu Aqoulah [23] found that some nurses turned off alarms or passed by frequent alarms without responding, potentially compromising patient safety. Furthermore, alarm fatigue and associated exhaustion may negatively affect moral responsibility and patient safety [4].

## 4. Discussion

The findings demonstrate the complex and multifaceted impact of alarms on intensive care nurses, including physical, psychological, and work‐related effects. Alarm fatigue appears to pose a significant threat to both psychological and physical well‐being.

Previous research indicates that a majority (75%–79%) of intensive care nurses experience stress and sleep disturbances related to alarms [27]. Anxiety, distress, and fatigue caused by alarm intensity and frequency may be associated with the negative effects of prolonged exposure.

Nurses with higher levels of work awareness, achieved through planning and structured workflows, appear to have greater tolerance and perceive alarms as less intrusive [28]. Education and training—particularly in specialist intensive care programs—may contribute to both psychological and physical strain. Bi et al. [29] demonstrated that alarm fatigue decreased following training in alarm management, including individualized alarm settings, which also reduced false alarms.

The majority of noise in ICU environments originates from alarms, which may contribute to an increased overall noise burden, particularly when alarms lack clinical relevance. Improved monitoring systems to reduce false alarms may be associated with improved efficiency and reliability. However, Drew et al. [30] found that nurses often rely on default settings rather than tailoring alarms to individual patient needs.

Alarms also interfere with nurses’ ability to perform essential tasks. The ICU environment has been described as a “cacophony” of sounds [31], which may contribute to frustration and a sense of inadequacy. Individualized alarm systems may reduce workload for nurses. Shanmugham et al. [32] found that default alarm settings were associated with higher workload compared to individualized settings.

Alarm fatigue may influence the act as a barrier, preventing nurses from performing their duties effectively. Given the already complex and demanding nature of intensive care nursing, alarm fatigue adds an additional burden that may compromise care quality.

Alarm fatigue also poses a significant threat to patient safety. Incidents involving disabled or ignored alarms have resulted in patient harm and death. Healthcare professionals have a legal and ethical responsibility to prevent such adverse events. However, the combination of high workload, complex care demands, and alarm fatigue creates a challenging environment for maintaining safe care.

Nurses reported decreased comprehensibility of their work environment, as structured processes became chaotic. A perceived lack of meaning and trust in alarm systems further contributed to reduced responsiveness. Frequent false alarms were associated with negatively affected response times, increasing the risk that critical alarms are misinterpreted or ignored [33, 34].

As medical technology continues to advance, the number of alarms is expected to increase. Consequently, the demands on intensive care nurses may intensify, highlighting the need for sustainable care practices and effective alarm management strategies.

Without clear strategies for managing alarms, there is a risk of negative health outcomes among nurses, including stress‐related conditions, which may ultimately compromise patient care.

This review contributes to the existing literature by synthesizing evidence on the multifactorial effects of clinical alarms on intensive care nurses. While previous reviews have primarily focused on alarm fatigue, the present review also incorporates findings related to nurses’ well‐being, cognitive workload, and work performance. By bringing together these aspects, the review provides a broader understanding of how clinical alarms influence both nurses and their work environment, highlighting considerations relevant to patient safety as well as occupational health.

### 4.1. Methodological Considerations

Daudt et al. [35] argue that the framework proposed by Arksey and O’Malley [9] is appropriate for scoping studies aiming to map the state of research within a given field. To capture the breadth of the existing knowledge base, both quantitative and qualitative studies were included in this review.

The search and selection process was conducted systematically and presented in a PRISMA (see Figure 1). Search terms were identified through initial exploratory searches and subsequently adapted to each database. Initially, studies including participants of all ages were considered; however, studies focusing exclusively on pediatric care were later excluded. This decision was based on differences in alarm design and functionality between pediatric and adult populations, which may affect the transferability of the findings. In the CINAHL database, the “All Adult” limiter was applied, as searches without this restriction yielded a large number of irrelevant results.

Attempts were made to identify subject headings and MeSH terms to strengthen the search strategy; however, these efforts did not yield relevant results. The authors were aware of their preunderstanding and the potential risk of bias this entails and therefore adopted a broad search strategy to reduce the likelihood of skewed findings. According to Polit and Beck [12], reliability is associated with the stability of data over time and under similar conditions. Broad search strategies were therefore considered essential to achieving a stable and comprehensive result. Several of the included studies utilized the same questionnaire to assess alarm fatigue [36], which strengthens reliability, although it also raises questions regarding the instrument’s validity in accurately measuring the phenomenon.

To enhance credibility, the software Rayyan was used to facilitate the screening process and enable independent assessments by the authors. Reference lists were also reviewed to ensure that no relevant studies were overlooked, thereby strengthening the study’s validity through increased data coverage.

The transferability of the findings is considered to be good within alarm‐intensive environments. Although the primary focus was on intensive care, the results may also be applicable to other settings, such as emergency departments, where monitoring equipment is frequently used.

In accordance with Arksey and O’Malley’s [9] methodology, the included studies were not subjected to formal quality appraisal in order to provide a broad overview of the existing knowledge. However, this approach may affect the overall credibility of the findings, as the methodological quality of the studies was not evaluated. Ethical considerations were addressed by examining whether the included articles reported informed consent and ethical approval. Most studies met these criteria, although one study lacked clear information regarding consent procedures, which should be considered when interpreting the results.

The analysis was conducted using manifest content analysis as described by Graneheim and Lundman [11], with an emphasis on remaining close to the data. Objectivity was sought by avoiding overinterpretation and allowing the data to speak for itself. To ensure validity, the articles were read multiple times, and relevant meaning units were identified and, when necessary, divided to avoid conflating multiple aspects. Color coding was used as a tool during the analysis process to further enhance structure and validity.

A potential limitation was that ethical considerations may not have been adequately addressed in all included studies. To assess the ethical foundations of the research, information regarding ethical approval and informed consent was reviewed. In most studies, authors reported either approval from an ethics review board or that participants had provided informed consent and were informed of their right to withdraw from the study at any time. In one study [25], only implied consent was reported, without a detailed description of the consent procedure. Nevertheless, this study was included due to its relevance to the research topic. This should be taken into consideration when interpreting the findings.

## 5. Conclusion

The findings of this study demonstrate that alarms significantly influence intensive care nurses’ daily work and overall health. Advances in medical technology, particularly in patient monitoring, have enabled more comprehensive surveillance of patients. However, this has also resulted in an increased number of alarms, contributing to a work environment characterized by frequent auditory stimuli.

It can therefore be concluded that alarms have a multifactorial impact on intensive care nurses, affecting their physical and psychological well‐being as well as their work performance.

### 5.1. Clinical Implications

The findings indicate that excessive alarms contribute to increased stress among intensive care nurses and reduce trust in alarm systems, suggesting a need for targeted interventions. One example of such an intervention is the use of alarm advisor systems.

In the study by Ruppel et al. [37], an alarm advisor is described as a software tool designed to assist nurses in maintaining awareness of the types and frequency of alarms triggered by individual patients. The system provides visual notifications on the monitor when repeated alarms for the same parameter are silenced within a specified time frame. Importantly, the system does not automatically adjust alarm thresholds but instead supports the nurse in making individualized adjustments.

More than half of the participating nurses reported a positive perception of the alarm advisor and found it helpful when setting alarm limits for patients. Additionally, the use of this tool was associated with a reduction in the total number of alarms.

## Author Contributions

All authors contributed to the conception of the study. Authors Frida Bråberg and Johan Vesterlund conducted the literature search and performed the study selection. All authors participated in the data analysis and the preparation of the manuscript.

## Funding

No funding was received for this manuscript.

## Conflicts of Interest

The authors declare no conflicts of interest.

## Supporting Information

Additional supporting information can be found online in the Supporting Information section.

## Supporting information


**Supporting Information** The supporting information includes two appendices. Appendix 1 provides the systematic search, including the search term combinations and the number of records retrieved. Appendix 2 presents an overview of the articles included in the analysis. The supporting information is available from the corresponding author (ME) upon reasonable request.

## Data Availability

The data that support the findings of this study are available upon request from the corresponding author. The data are not publicly available due to privacy or ethical restrictions.
