# Supplementary material for: The Impact of Frequent Alarm Exposure on Intensive Care Nurses—A Mapping of the Current State of Knowledge
Source: Crit Care Res Pract. 2026 Nov 4;2026:3865004. doi: 10.1155/ccrp/3865004 (PMC13732415; doi:10.1155/ccrp/3865004)
Supplement: Supplementary file 1 [file CCRP-2026-3865004-s001.zip › Corrigert II Appendix II Overview of articles.docx]

**Overview of articles**

| Author, Year, Country | Studie design | Aim of the Studies | Method | Selection | Results |
| --- | --- | --- | --- | --- | --- |
| Alkubati, S. A., Alsaqri, S. H., Alrubaiee, G. G., Almoliky, M. A., Alqalah, T. A. H., Pasay-An, E., Alrasheeday, A. M., & Elsayed, S. M. (2024) Egyptian | Cross-sectional study | The aim of this study was to evaluate the level of alarm fatigue and determine the relationship between sociodemographic and work-related factors that may influence alarm fatigue in intensive care units in Saudi Arabia.. | Torabizadeh's survey (AFS) | All hospitals in Hail City. 400 intensive care nurses were given a survey, 298 completed it (75% response rate) | The overall mean alarm fatigue score was 26.38±8.30 out of 44. The highest score was observed for the question “I pay more attention to alarms in certain situations”, while the lowest score was observed for the questions “I turn off alarms at the beginning of each work shift”, with mean scores of 2.51 and 1.61, respectively. |
| Bani Hani S & Abu Aqoulah E A (2024)  Jordan | Cross-sectional study | The study aims to assess the level of alarm fatigue and stress among intensive care nurses | Torabizadeh's online survey and perceived stress scale (PSS) | Five hospitals 127 intensive care nurses | Intensive care nurses showed significant alarm fatigue with a mean score of (M = 30.1 ± SD = 7.47). A moderate level of stress (M = 21.5 ± 5.02). |
| Carellia L, Terzonia S, Destrebecqb A, Formentic P, Soumahorod F, Espositoe A & Ferrara P. (2021) Italie | Cross-sectional study  Multicenter study | S The aim of the study was to evaluate the psychometric properties of the Alarm Fatigue Questionnaire and to investigate the phenomenon of alarm fatigue among nurses working in different intensive care units in Italy. | Torabizadeh's survey (Italian translation) | 396 (79.84% response rate) nurses from four large Italian hospitals. | The results from the survey showed a mean value of 29 [22;30] for alarm fatigue. 42.17% of the sample reported that incidents have occurred in connection with alarm fatigue. |
| Dehghan M, Mokhtarabadi S, Rashidi E, Rahiminejad E & Asadi N (2023) Iran | Cross-sectional study | The aim of this study was to determine the relationship between quality of work life and burnout syndrome among nurses in intensive care units.. | Data were collected via three questionnaires (sociodemographic, ProQOL and a questionnaire [AFS]) | Three hospitals 201 intensive care nurses | 257  The mean scores for empathic satisfaction (CS), burnout, secondary traumatic stress (STS), and AFS were 34.66 ± 7.54, 28.98 ± 7.59, 27.69 ± 5.87, and 13.48 ± 7.32. The majority had no or mild AFS, moderate CS, burnout, and STS. |
| Gül, I &. Soyer Er Ö. (2025)  Turkey | Cross-section study | The aim of the study was to determine the mediating role of moral courage and the relationship between alarm fatigue and patient safety climate among nurses in surgical intensive care units and operating rooms. | Torabizadeh's survey  Nurses moral courage scale  Patient safety scale | 268 nurses  2 hospitals | The result showed 15.45 ± 6.20 for  alarm fatigue.  It showed that nurses experienced alarm fatigue to a moderate degree, civil courage to a high degree and the experience of patient safety to a moderate degree. |
| Honan, L., Funk, M., Maynard, M.,Fahs, D., Clark, T., &  David, Y. (2015)  USA | Quantitative | To qualitatively explore nurses’ experiences  with clinical alarms. | An open-ended survey question | Nine national organizations supported the parent study by posting links to the online survey on their respective websites.  406 nurses | Six interrelated themes emerged: dissonance  and desensitization; pollution, panic, and pathology;  calling for accountability; calling for authority of nurses;  clinical alarm management is crucial but not a panacea;  and hope for the future. Six interrelated themes emerged: dissonance  and desensitization; pollution, panic, and pathology;  calling for accountability; calling for authority of nurses;  clinical alarm management is crucial but not a panacea;  and hope for the future. |
| Karahana A, Kava S, Cevik B, Çıtak E A,  Uğurlu Z & Fulserb B, (2023)  Turkey | Descriptive study | The purpose of this study was to find out the experience of alarm fatigue among nurses working in the ICU. | Questionnaire | University hospitals in five cities  592 nurses (74% response rate) | More than half of the nurses experienced problems with alarms, especially false alarms. Alarm fatigue decreased with age and number of years in the profession. |
| Lewandowska, K., Weisbrot, M., Cieloszyk, A., Mędrzycka-Dąbrowska, W., Krupa, S., & Ozga, D. (2020)  Polen | Systematic literature review | The purpose of the study is to review the available literature on nurses' perceptions of clinical alarms and their impact on work in the intensive care environment. | PRISMA guidelines for systematic reviews. | 7 articles were selected for analysis and results | The results show that 93% of the nurses included felt that alarm fatigue can lead to alarms being silenced or ignored. 81% of respondents felt that alarm fatigue stems from excessive false alarms. |
| Nyarko, B. A., Yin, Z., Chai, X., & Yue, L (2023)  Ghana | Cross-sectional study | The aim of this study was to investigate the degree of alarm fatigue and its associated factors, as well as to determine its relationship with burnout among nurses working in intensive care units in hospitals in Ghana. | Three methods were used. One method is based on a theoretical framework, the second is a questionnaire that assesses alarm fatigue, the third is a tool called MBI (Maslach Burnout Inventory). | Five hospitals in Ghana. 365 nurses | The study showed an average alarm fatigue score of 76.43 ± 27.80 out of a maximum of 124. |
| Salameh B, Abdallah J, Alkubati S A & Albashtawy, M (2024)  Palestine | Descriptive cross-sectional study | The aim of this study is to investigate alarm fatigue and stress levels among intensive care nurses. In addition, the study aims to assess factors that predict both alarm fatigue and perceived stress. | Torabizadeh online survey and perceived stress scale (PSS) | 187 out of 251 intensive care nurses responded to the survey. | The average total alarm fatigue score was 23.36 (SD = 5.57) out of 44. The study showed that 62.6% of participating intensive care nurses experience an average to high degree of alarm fatigue, while 69.5% experience average to high levels of perceived stress. |
| Salous, M., Alkhawaldeh, J., Kewan, S., Aburashideh, H., Bni Hani, D., & Alzayyat, A. (2017) USA | Systematic literature review | The aim of this review was to examine critical care nurses' attitudes related to alarm fatigue. | Summary results from the included articles | The selected articles included those published between 2002 and 2016, as well as those that contained information on historical background related to alarm fatigue and its impact on patient safety.  50 articles were selected that were considered useful to support the study. | The review showed that alarm fatigue caused nurses to not act on alarms due to excessive false alarms and alarms that do not require action, leading to sensitization and sensory overstimulation. |
| Şen, H.T., Bilgin, O., Alan, H., & Polat, Ş. (2024)  Turkey | Descriptive cross-sectional study and correlational design | The aim of the study was to assess the levels of alarm fatigue and empathy fatigue among intensive care nurses, and to investigate whether empathy fatigue is linked to alarm fatigue and whether it can predict its occurrence. | Torabizadeh's questionnaire  PROQOL scale | 413 nurses | The result showed an alarm fatigue of 23.05 ± 6.10 (min = 6, max = 42), which shows a moderate level  For empathy fatigue the result showed 21.25 ± 11.59 (min = 0, max = 60), which shows a level just below moderate. |
| Storm, J. & Chen, H.C. (2020)  USA | Observational study | The study investigates how various factors, such as emotional stress, working conditions and personal characteristics, affect alarm fatigue in intensive care nurses and whether these factors can predict alarm fatigue. | Strengthening the Reporting of Observational Studies in  Epidemiology (STROBE) checklist.  Data were collected using observations, ProQOL, demographic surveys. | 8 intensive care units.  4 step-down units.  52 nurses. | 40% of nurses showed signs of alarm fatigue and were at risk for burnout and empathy fatigue. There was no significant relationship between alarm fatigue and empathy fatigue, burnout or empathic satisfaction. However, there was a significant relationship between alarm fatigue and women, age 21–40 years. |
