# Supplementary material for: The Impact of Frequent Alarm Exposure on Intensive Care Nurses—A Mapping of the Current State of Knowledge
Source: Crit Care Res Pract. 2026 Nov 4;2026:3865004. doi: 10.1155/ccrp/3865004 (PMC13732415; doi:10.1155/ccrp/3865004)
Supplement: Supplementary file 1 [file CCRP-2026-3865004-s001.zip › Corrigert II Appendix I Systematic search.docx]

**Systematic search**

| **Database**  **Cinahl 20250203** | **Key words** | **Hits** |
| --- | --- | --- |
| #1 | ”icu or intensive care unit or critical care” | 208,257 |
| #2 | “nurses experience or perspective or view or attitude” | 1,180,957 |
| #3 | “alarm fatigue” | 441 |
| #4 | “alarm burnout or alarm monitor or alarm system” | 2,284 |
| #5 | S1 AND S2 AND S3 AND S4 | 62 |
| Delimitation: | English language, published 2015-2025, All adult | 15 |

| **Database**  **PubMed 20250203** | **Key words** | **Hits** |
| --- | --- | --- |
| #1 | “Alarm fatigue” | 663 |
| #2 | ”icu” | 199,405 |
| #3 | “critical care” | 449,754 |
| #4 | “(icu) OR (critical care)” | 574,685 |
| #5 | “nurses experience” | 64,138 |
| #6 | “nurses perspective” | 22,215 |
| #7 | “nurses view” | 6,903 |
| #8 | “nurses attitude” | 84,932 |
| #9 | “(((nurses experience) OR (nurses perspective)) OR (nurses view)) OR (nurses attitude)” | 143,889 |
| #10 | “((alarm fatigue) AND ((icu) OR (critical care))) AND ((((nurses experience) OR (nurses perspective)) OR (nurses view)) OR (nurses attitude))” | 51 |
| Delimitation: | English language, In the last 10 years, Free full text | 21 |

| **Database**  **Psychinfo 20250203** | **Key words** | **Hits** |
| --- | --- | --- |
| #1 | “Alarm fatigue” | 104 |
| #2 | “nurse experience or nurse perspective or nurse view or nurse attitude” | 49,900 |
| #3 | “icu or critical care unit” | 8,839 |
| #4 | S1 AND S2 AND S3 | 9 |
| Delimitation: | published 2015-2025 | 9 |

| **Database**  **Cochrane**  **20250203** | **Key words** | **Hits** |
| --- | --- | --- |
| #1 | “Alarm fatigue” | 0 |

| **Database**  **PubMed 20260917**  **Supplementary search** | Keywords | Hits |
| --- | --- | --- |
|  | “((alarm fatigue) AND ((icu) OR (critical care))) AND ((((nurses experience) OR (nurses perspective)) OR (nurses view)) OR (nurses attitude))” | 87 |
| Delimitation | Published between 2015 -February 2025 | 67 |
| Delimitation | Published between 2015 -February 2025 English language | 49 |
|  |  |  |
